# Assessment of Clayey Freshwater Sediments as Suitable Precursors for Alkaline Activation

**DOI:** 10.3390/polym16020175

**Published:** 2024-01-07

**Authors:** Jan Fořt, Ayodele Afolayan, Martin Mildner, Petr Hotěk, Martin Keppert, Robert Černý

**Affiliations:** Department of Materials Engineering and Chemistry, Faculty of Civil Engineering, Czech Technical University in Prague, Takurova 7, 166 29 Prague, Czech Republic; ayodele.afolayan@fsv.cvut.cz (A.A.); martin.mildner@fsv.cvut.cz (M.M.); petr.hotek@fsv.cvut.cz (P.H.); martin.keppert@fsv.cvut.cz (M.K.); cernyr@fsv.cvut.cz (R.Č.)

**Keywords:** alkali-activated material, freshwater sediment, porosity, particle size, mechanical strength

## Abstract

One of the biggest challenges in the construction industry in recent times is the mitigation of the environmental impact of this sector, the reduction in dependence on primary raw materials, and the reduction in CO_2_ production while maintaining functional properties. Alkaline activation of a number of waste products represents a promising way to achieve the above-mentioned goals, but the availability of a number of waste products changes over time, especially in Europe. While freshwater sediments were in the past widely utilized as an agricultural fertilizer, recent precautions have significantly decreased such application, and thus new destinations must be delivered. To explore the potential of freshwater sediments, select samples from various locations were subjected to detailed characterization to verify the applicability of the material for alkali activation. As recognized, the selected sediments contain a substantial volume of desired mineralogical compounds that can serve, after 900 °C curing, as suitable precursors. Such samples have consequently activated the mixture of alkaline activators to obtain dense structures and were subjected to detailed investigation aimed at understanding the mechanical parameters. The obtained mechanical results ranging between 14.9 MPa and 36.8 MPa reveal the engineering potential of sediments for valorization through alkali activation and outline new research challenges in this area.

## 1. Introduction

Portland clinker production and the concrete industry are currently facing significant challenges due to carbon dioxide restrictions that must be met in the upcoming decades. Measures such as the Carbon Border Adjustment Mechanism (CBAM), meeting the requirements of the Fit for 55 and carbon-zero economy until 2050 significantly influence the future of these industries due to their significant contribution to global emissions production [1,2,3]. Measures that can significantly reduce these emissions include redefining the material base of the construction industry and replacing Portland cement with another material. In this regard, significant progress has been achieved in recent years in the field of alkali-activated materials research, which has enabled a complete replacement of Portland clinker and, thus, significant environmental savings [4,5,6].

Alkali activation of various waste materials has been touted as a possible solution to the issue of the energy intensity of traditional binders’ production, increased costs, and reduction in waste generation [7,8]. Facing environmental issues, cement production requiring exposure to high temperatures around 1,400 °C and excessive release of carbon dioxide to the atmosphere during the thermal decomposition of calcium carbonate represent processes that need to be minimized and replaced by more energy-efficient alternatives [9,10,11].

Alkali-activated materials (AAMs) or geopolymers are generally obtained by the interaction between the aluminosilicate precursor in the powdered form and highly alkaline solution usually based on sodium hydroxide and silicate. The reaction between silica-alumina-rich precursors and alkali solution results in the formation of aluminosilicate binder gel with similar physical properties to those of hydrated cement but with different chemical properties and reaction mechanisms [6,12]. AAMs typically have amorphous to semi-crystalline microstructures, and during the alkali-activation process, there is not enough time and space for the gel to arrange into a crystalline framework; therefore, the resulting product is amorphous or semi-amorphous in nature [7,13]. AAMs’ properties are influenced by the type and amount of alkali activator used, the size and shape of the precursor, and reaction conditions [14,15,16,17].

Since various wastes are generated from industries on a daily basis, different types of materials and industrial by-products with amorphous characteristics and divergent ability to release a reasonable amount of silica-alumina for alkali-activation reactions are being employed in the production of alkali-activated materials (AAMs) [14,18]. In this sense, materials such as metakaolin (MK) [19], coal fly ash (FA) [20], and blast furnace slag (BFS) [21] have been extensively studied as promising alternatives to Portland cement (PC), as the alkali activation of these by-products may provide significant benefits over only partial PC replacement resulting from complete cement replacement. Specifically, low-calcium fly ash (class F) is commonly used as a major precursor in AAMs due to its availability and potential for better performance [22]. It offers longer setting times and improved workability compared to high-calcium fly ash [23]. BFS is another industrial by-product generated during the production of iron and steel. It has a silica–alumina ratio ranging from 1.71 to 3.67 and contains calcium oxide [24]. BFS can be utilized as a sustainable alternative to cement, as it improves porosity, long-term strength, and resistance to chemical attack, and reduces water demand and permeability. Some other suitable industrial by-products are waste glass powder (WG), paper sludge, mine tailings, coal gangue (CG), high-magnesium nickel slag (HMNS), marble dust, volcanic ash, and waste catalyst residue discharged from various industrial processes [25]. However, besides technical feasibility and performance, several other factors must be considered for an overall assessment of the viability of this approach. In general, the limited availability of the most used precursors such as FA and BFS in Europe poses a main constraint to the future development of such material for the building practice [19,26].

Water reservoirs can be deemed as abundant sources of freshwater sediments (FWSs), which have to be dredged to extend and increase the capacity of reservoirs [27]. FWSs consist of a variety of elements, such as organic matter and aluminosilicate minerals like quartz, feldspar, illite, smectite, chlorite, and kaolinite, which have a great potential for construction materials [28,29,30]. Large quantities of sediments accumulate in rivers and dams worldwide, and they are solid particles that are produced by movement in the water flow or deposited by wind and gravity [31]. The huge volume of sediments is harmful to the environment and human society in the form of debris flow, flooding, and pollution. They cause reservoir siltation, which can affect the flood capacity of rivers, navigation efficiency, and water-conservation projects. Also, large amounts of sediment accumulated after dredging will not only cause a waste of land resources but will also cause ecological problems [31,32,33].

However, despite the wide availability and favorable chemical composition of FWS, their utilization faces significant challenges due to the limited proportion of an amorphous phase, which can result in low material reactivity [28]. Although this perception has been challenged by several researchers, pioneering studies by [13,34] have demonstrated satisfactory functional parameters. Similarly, the notion of highly amorphous precursors was overcome [7]. Pretreating FWS can increase the content of the reactive amorphous phase from an initial 18 wt.% to 51 wt.%, making it a highly suitable precursor for AAMs production [35,36]. However, quite a wide range of applied temperatures as described in the literature range from (400 °C to approximately 1085 °C), which did not provide consistent knowledge, and several gaps can be identified [28]. It was demonstrated that strong polymer bonds are formed when enough Si and Al ions are solubilized during the alkali activation of FWS [37]. Albeit, these findings have not been thoroughly examined in conjunction with recent knowledge on the alkali activation of low amorphous precursors. Additionally, the challenge of achieving the appropriate chemical composition and balanced molar ratios as well as determining the optimal molarity of applied activators remains a significant hurdle in the effective utilization of this resource [23].

The reassessment of the current material base used in the construction industry represents a significant turning point in development. At the same time, it represents a significant challenge that must be overcome in terms of finding and valorizing suitable and available raw materials. The use of Portland cement, slag, as well as coal fly ash as basic binding materials in Europe will not be possible in the future in terms of their price or availability. The principles of circular economy and sustainable development emphasize the valorization of locally obtained raw materials, which currently do not undergo adequate use and will possess significantly limited disposal from 2035. Most contemporary research in this area focuses on classical precursors; however, freshwater sediments, which may show certain similarities to clay materials, remain neglected and their potential in this regard is not studied. The proposed research aims to address these challenges by investigating the utilization of freshwater sediments produced across Europe in large volumes with a lack of disposal scenarios to access a viable method of valorization. In this regard, eight types of FWS are studied to understand their chemical and mineralogical nature. Through the employment of elevated temperature treatment, their potential for alkali activation and the formation of the alkali-activated structure is assessed, including the determination of their mechanical performance.

## 2. Materials and Methods

### 2.1. Raw Materials

This research work is limited to the production of alkali-activated materials using FWS collected from different reservoirs in the Czech Republic. One of the main challenges in utilizing FWSs is the limited amount of amorphous phase, which translates to lower material reactivity [28]. However, pretreating FWS at elevated temperatures enhances its reactivity through polycondensation reaction [35]. A temperature of 900 °C based on preliminary experiments was employed for the pretreatment of the dried samples. The calcination process was conducted using a Classic Clare 4.0 furnace (Prague, Czech Republic) at a heating rate of 5 °C/min for 3 h which was maintained at a constant temperature of 900 °C for another 3 h. After calcination, the materials were cooled in ambient air and ground with a ball mill machine for 1 h at a rotational speed of 6 rpm to obtain fine powder samples. The samples were subsequently sieved using a 0.125 mm sieve mesh. The FWSs were labeled as A1–A8 before calcination, and A1C–A8C after calcination.

### 2.2. Samples Preparation

For the alkaline activation of the calcined FWSs, a mixture of sodium hydroxide NaOH and potassium silicate solution K_2_SiO_3_ was used. The potassium silicate solution was provided by Vodní sklo, Czech Republic, with a molar ratio SiO_2_/K_2_O = 3. Sodium hydroxide solutions 8M were prepared by dissolving NaOH pellets (Merck KGaA, Darmstadt, Germany, purity ≥ 98%) in distilled water and storing them to cool to room temperature.

The components were further homogenized using a FORM +TEST Prufsysteme mixer (FORMTEST, Riedlingen, Germany) at a speed of 20 rpm for 10 min until gelation occurred. The water dosage used to obtain the same rheologic properties was adjusted using by flow table test (diameter = 180 mm). The resulting paste was poured into molds in three replicates, and the solidified samples were unmolded after 48 h. The unmolded samples were aged under ambient temperature conditions for 28 days from the day of mixing and subsequently dried at 80 °C for 2 days to obtain steady-state weight before mechanical tests. The physical properties, mass, and bulk density were measured, and compressive strength and flexural strength tests were performed using the destructive strength test method. Details of the mixture compositions are provided in Table 1, where individual mixtures are denoted from FWS1 to FWS8 according to the used sediments (from A1C to A8C).

### 2.3. Employed Experimental Setup

A laser diffraction measuring device with a measuring range of up to 2 mm was used for the determination of the particle size distribution of the applied precursors. Large particles were detected using an infrared laser with a large distance to the measuring cell, and for small particles a green laser with a small distance to the cell was used, which permitted the detection of forward scattered light up to a scattering angle of 65°. The measurement of the smallest particles down to the nano-range was performed by the green laser light for backward scattering.

The chemical composition of the obtained sediments was determined by X-ray fluorescence (XRF) by using the device Thermo ARL 9400 XP (Thermo Fisher Scientific, Darmstadt, Germany). The reproducibility of the measurement was 0.0001% and the standard measurement error was 0.02%. The phase composition was examined by an X-ray diffraction (PANalytical X’PertPRO (Malvern Panalytical Ltd., Malvern, UK) diffractometer equipped with a CoKa X-ray tube (40 kV, 30 mA). The data were evaluated using the HighScorePlus software package (version 3.0.5) and JCPDS PDF2 database. The Rietveld analysis was performed using Topas software to determine the content of crystalline phases. The samples for XRD analysis were pulverized using agate mortar to pass a 20 mm sieve. The quantification of the amorphous portion was performed with the help of an internal standard (ZnO, 10%).

Among the basic physical properties of studied AAMs, the bulk density, matrix density, and open porosity were measured. The bulk density was determined by weighing of the samples with a known volume obtained using a digital caliper. The matrix density was obtained by using a helium pycnometer Pycnomatic ATC (Thermo Fisher Scientific) device. Consequently, the total open porosity was calculated on the basis of the knowledge of bulk and matrix density.

Mercury intrusion porosimetry (MIP) analysis was conducted to characterize the inner structure of studied materials. Pore size distribution was measured using a combination of porosimeters: Pascal 140 and Pascal 440 (Thermo Fisher Scientific). During the evaluation of measured data, the circular cross-section of capillaries was assumed, whereas the mercury contact angle was assumed to be 130°.

A hydraulic testing device VEB WPM Leipzig (Leipzig, Germany) equipped with a stiff loading frame with a capacity of 3000 kN was employed for the determination of compressive and bending strength. Flexural strength was tested according to CSN EN 12390-5. For these measurements, prismatic samples with dimensions of 40 mm × 40 mm × 160 mm were used. The compressive strength was measured in line with CSN EN 12390-3 on the portions of prisms broken in the bending test; the loading area was 40 mm × 40 mm.

## 3. Results and Discussion

### 3.1. Materials Characterization

The phase composition of the raw sediments was studied by powder X-ray diffraction. The results are presented just semi-quantitatively (Table 2) since exact quantification in systems containing three clay minerals in the mixture is very difficult, if not impossible. The main components of all sediments were clay minerals (chlorite, illite, and kaolinite in varying proportions) accompanied by quartz. Further, all three sediments contained some feldspars and a small amount of hornblende (dark pyroxene with origin in the parent material). Interestingly, the presence of hillebrandite (hydrated calcium silicate Ca_2_(SiO_3_)(OH)_2_) was found, and it is possibly a weathering product of a parent material.

**Figure 1:**
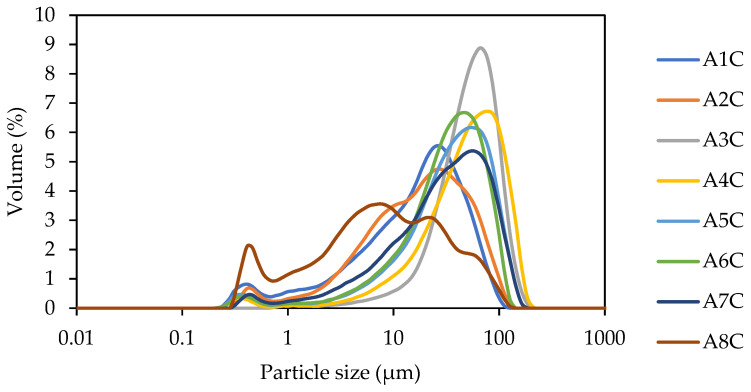
Particle size distribution of selected freshwater sediments.

Particle size distribution (PSD) of sediments after thermal treatment and grinding provides insights into a material’s behavior, and it can be used to optimize the material composition to improve its workability and strength. It determines the range of particle sizes in the material and influences the packing density, reactivity, and permeability of the material. The provided PSD results indicate significant differences between particular materials. The average size distributions d50 varied from 27.22 µm to 50.78 µm as depicted in Figure 1. Except for sample A8C, all curves show only a single-peaked maximum that ranges in the above interval. Sample A8C shows two or three main peaks, which can be identified around 0.7 µm, 9 µm, and 20 µm. In this sense, all materials can be compared with traditional binders with a main peak of around 50 µm [38].

The chemical composition of calcined sediments was examined by XRF spectroscopy (Table 3 and Table 4). There were not any significant differences between the individual studied sediments; their chemical composition corresponded to what one may expect from the clayey freshwater sediments. Before material characterization in a hardened state, all samples were dried to remove excessive moisture content and consequently characterized by the set of experiments. The majority of FWSs formed by SiO_2,_ followed by a distinct portion of Al_2_O_3_. The content of hazardous materials reached a similar level to EU fly ash for most of the recognized elements [39]. Here, only samples A7C a and A8C exhibited slightly higher concentrations of Mn and Zn compared to other materials. The obtained data correspond with other studies [40]; however, the limits for their potential application in the construction industry are not correlated with consequences in the aquatic environment. This section may be divided by subheadings. It will provide a concise and precise description of the experimental results, their interpretation, as well as the experimental conclusions that can be drawn.

**Figure 2:**
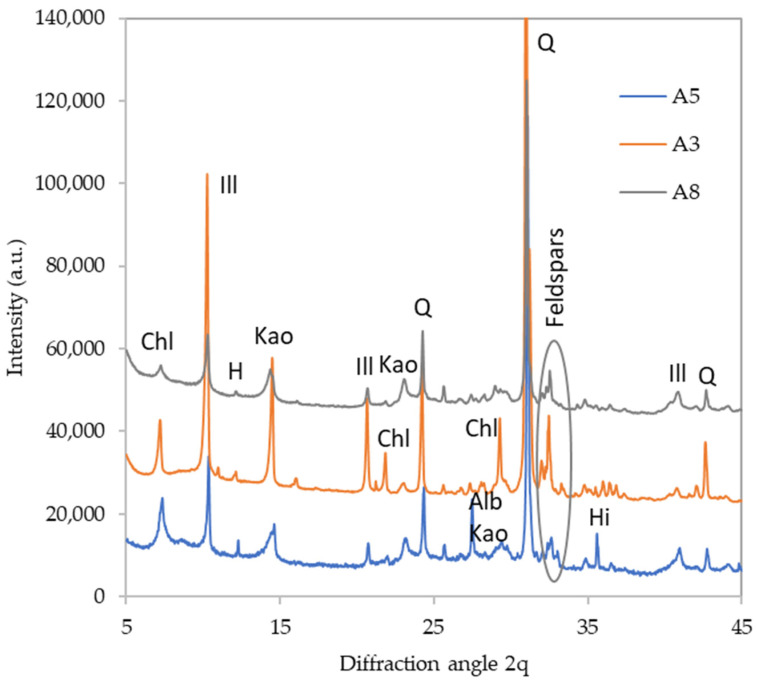
Phase composition of raw sediments. Chl—chlorite, Ill—illite, H—hornblende, Kao—kaolinite, Q—quartz, Alb—albite, Hi—hillebrandite.

**Figure 3:**
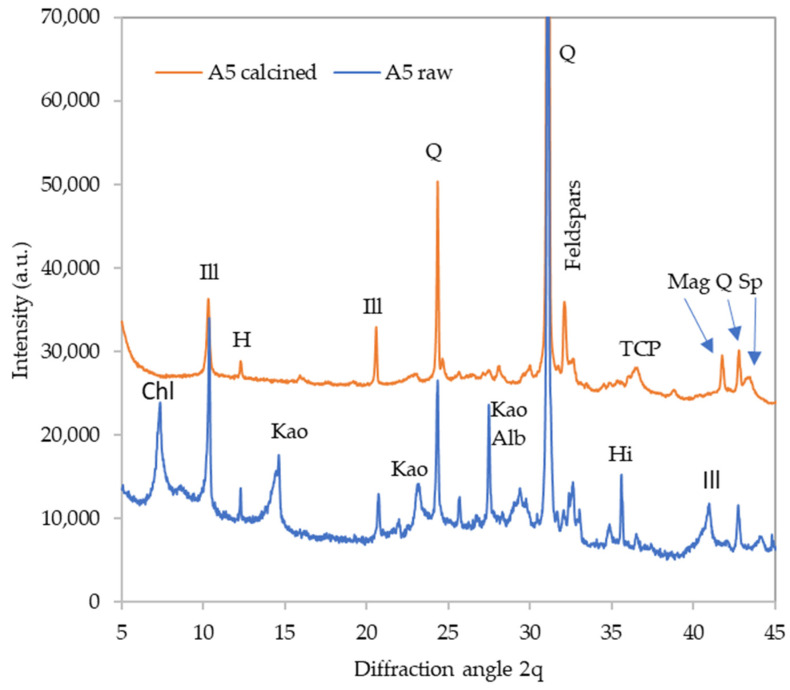
Phase composition of raw and calcined sediment A5. Chl—chlorite, Ill—illite, H—hornblende, Kao—kaolinite, Q—quartz, Alb—albite, Hi—hillebrandite, TCP—tricalcium phosphate, Mag—maghemite, Sp—spinel phase.

**Figure 4:**
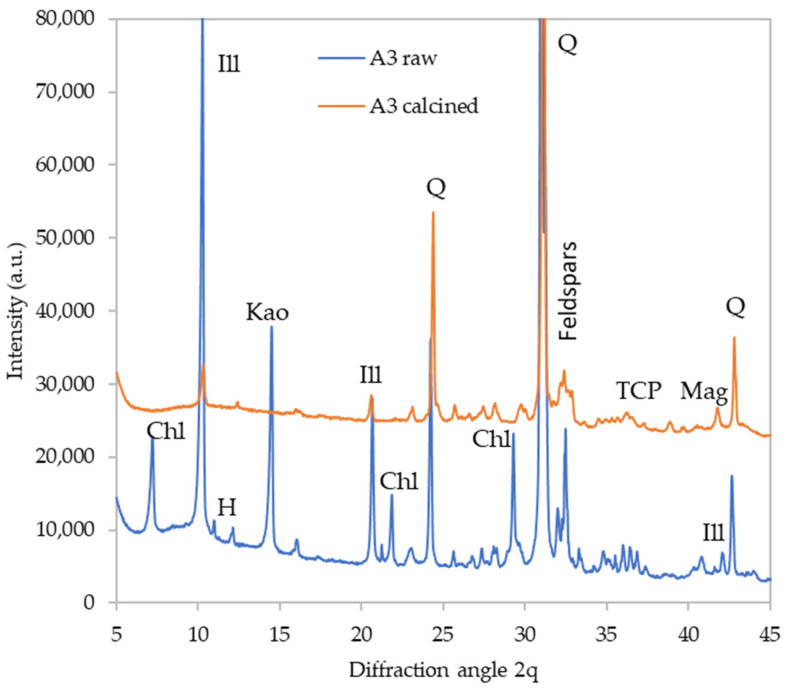
Phase composition of raw and calcined sediment A6. Chl—chlorite, Ill—illite, H—hornblende, Kao—kaolinite, Q—quartz, TCP—tricalcium phosphate, Mag—maghemite.

**Figure 5:**
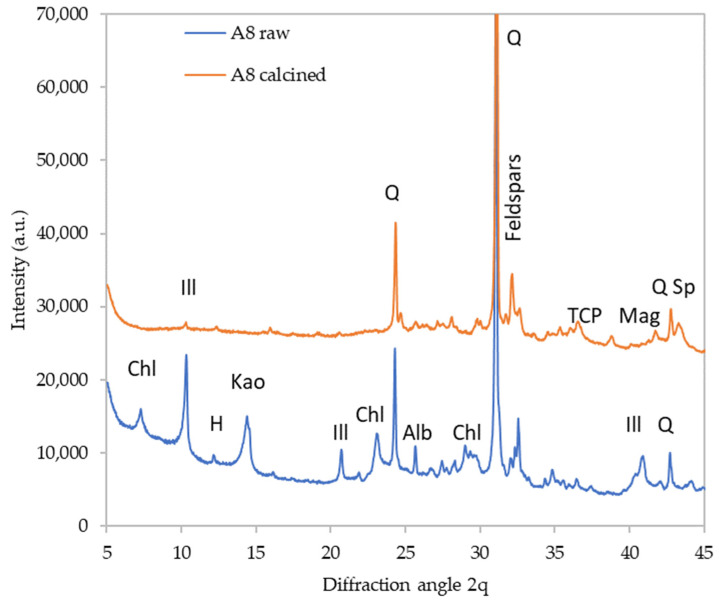
Phase composition of raw and calcined sediment A8. Chl—chlorite, Ill—illite, H—hornblende, Kao—kaolinite, Q—quartz, TCP—tricalcium phosphate, Mag—maghemite, Sp—spinel phase, Alb—albite.

The detailed diffractograms of selected materials are provided in Figure 2, Figure 3, Figure 4 and Figure 5 to depict the most distinct differences. The effect of calcination on the phase composition may be observed by comparing the X-ray diffractograms of raw and calcined sediments. The quantitative results are summarized in Table 5. Figure 3illustrates the changes that took place in sediment A5. The calcination caused dehydroxylation in all present clay minerals; kaolinite and chlorite crystals were decomposed completely while some fragments of illite structure were preserved even at 1000 °C. The clay minerals’ decomposition was the source of the amorphous phase in the calcined samples. Moreover, the calcination caused the decomposition of hillebrandite (calcium silicate hydrate). On the other hand, calcination promoted the crystallization of new phases; the present phosphate anions crystalized as Ca_3_(PO_4_)_2_ (TCP). Iron-bearing structures present in the raw sediment crystalized as maghemite (γ-Fe_2_O_3_) and a spinel phase appeared as well. The sediment A3 underwent a similar transformation (Figure 4), while the sediment A8 seemed to be somewhat more amorphized, which is especially apparent from the very-low diffractions of mica (illite) residuals as well as the lower content of feldspars (Figure 5).

### 3.2. AAMs Characterization

**Figure 6:**
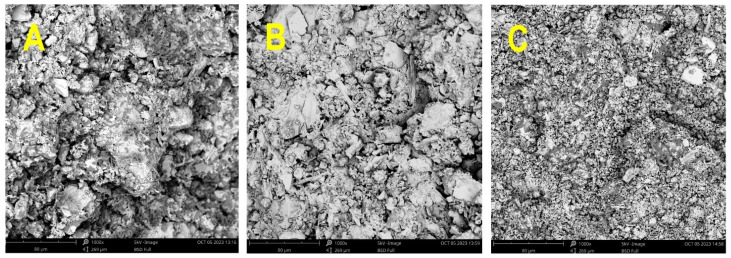
SEM images of (**A**)—FWS5, (**B**)—FWS3, (**C**)—FWS8.

Figure 6 provides insight into the differences in the microstructure of formed alkali-activated FWS. Samples are listed in order from least amorphous to most amorphous, namely, FWS5 (precursor A5C) to FWS3 (precursor A3C) and FWS8 (precursor A8C) samples. The matrix of FWS8 specimens is notably more homogenous, indicating a higher reactivity degree of the raw material compared to FWS5 based on visual observation and the occurrence of cracks and crystal formations. According to the content of the amorphous phase, the structure depicted in Figure 6A,B seems to be more rugged, with distinct cracks. In this sense, only minor differences can be observed between 6a and 6b. The observed cracks can be attributed to the loss of water during the hardening period and consequent volumetric changes.

To provide the basic characteristics of the alkali-activated FWSs in a hardened state, the bulk density, matrix density, and total open porosity were determined. As one can see from the summarized results provided in Table 6, the bulk density values varied from 1.64 g/cm^3^ to 1.89 g/cm^3^ and matrix density from 2.47 g/cm^3^ to 2.56 g/cm^3^. The total open porosity (TOP) varied to a greater extent, namely from 24% to 35%, indicating that select materials have more interconnected voids accessible from the external surface. In structural applications, a low TOP is often desired to ensure adequate mechanical strength, durability, and resistance to moisture ingress. However, the correlation between TOP and mechanical performance for AAMs differs from concrete due to more complicated hydration mechanisms and the formation of various structures [7,41]. The role of the TOP cannot be deemed as crucial since the chemical and mineralogical compositions, including the role of particle size, were found to be more important. Notwithstanding, this contribution to the understanding of the water and heat transmission properties represents the key parameter [42].

**Figure 7:**
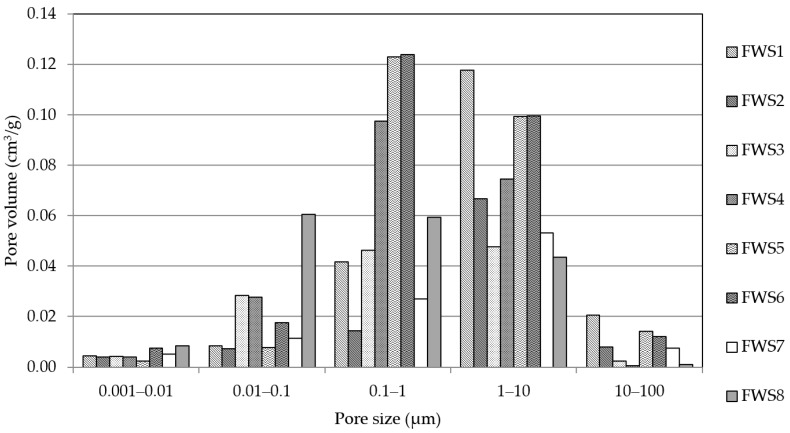
Pore size distribution of studied materials.

Porosimetry analysis helps in optimizing the properties of AAMs in general by controlling the pore structure. Information about the specific pore volume, a significant parameter representing the collective volume of pores in the material, helps in understanding the pore structure distribution and porosity within the material. Figure 7 represents the distribution of pore diameters of the studied materials. Looking at the provided results, one can see a major difference in the pore size distribution and the correlation with the mineralogical composition of the used precursors. While the precursors with a lower portion of amorphous phase exhibit larger pore size, especially in the range of 0.1–1 µm and 10–100 µm, precursors with an amorphous portion share of 10–18% formed a denser structure with reduced pores in the above-mentioned ranges. This effect is more pronounced for mixture FWS8, which has a significantly higher content of amorphous phase. Here, a notable shift in the pore volume in the range of 0.01–0.1 µm can be observed. At a larger pore diameter of 10 µm and above, the recorded pore volume essentially approaches zero, and a flat slope was observed, which signifies the absence of larger pores in the materials. The correlation between amorphous composition and bulk composition has been previously mentioned by other authors [42,43], who found that pore radius is a critical parameter for understanding the durability of materials. Moreover, the role of the amorphous phase and the molar ratios was also confirmed [44]. In addition to the silicate content, the molar ratios play a vital role in the formation of the dense structure and mechanical properties in the hardened state [45,46,47]. The sensitivity of the transition pores and the overall creation of larger voids in the material structure can be related to the concentration of alkalis within the activation period and the Si/Al ratio [48]. However, recommendations for the ideal composition are different for individual precursors and cannot be universally adopted.

**Figure 8:**
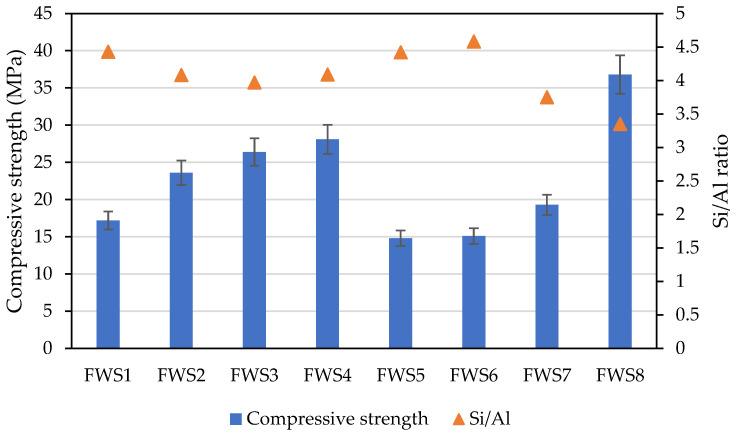
Compressive strength of alkali-activated freshwater sediments.

Figure 8 illustrates the compressive strength of the designed materials after 28 days of wet curing. The recorded compressive strength ranges from 14.59 MPa to 37.09 MPa across the samples. These values can be compared to C35 concrete, making them suitable for potential future structural applications. The highest level of compressive strength can be assigned to sediment A8C, which has the highest portion of the amorphous phase despite having an overall higher level of total open porosity. On the other hand, the lowest mechanical performance belongs to sediments A1C, A5C, and A6C (mixtures FWS1, FWS5, and FWS6) with amorphous content below 10%. Also, the relation to the particle size and Si/Al ratio can be observed as follows. Sediment A8 resulted in the lowest particle size diameter of 7.22 µm and a multimodal particle distribution curve which corresponds to the highest mechanical strength of 36.09 MPa despite the slightly higher dosage of extra water. This phenomenon also refers to the beneficial effect of finer particles and higher surface area with a better reactivity over coarser particles [38,49]. The slightly lower particle size of sediments A2C and A3C may be viewed as a beneficial parameter for hardened structure formation. In comparison with the values of the compressive strength introduced in the work of Komnitsas [27], which reached a compressive strength between 19 and 24 MPa after using KOH as a primary activator of marine sediments, the provided results correspond with these values, with the exception of the FWS8 mixture. Here, the slightly higher content of amorphous silicate contained in the potassium water glass improved the reactivity of precursors thanks to the better solubilization and formation of aluminosilicate bonds [50]. In addition, the high dosages of NaOH may lead to the depolymerization of formed products and consequent weakening of the material microstructure as described in the work of Ye et al. [51]. In addition to the interaction between Na^+^ and K^+^ ions with Si-OH and Al-OH, the Si/Al molar ratio is deemed as an essential factor for phase formation; however, curing conditions have to be taken into account too. In this sense, further improvement in the mechanical properties can be attained by the employment of an increased curing temperature that promotes the reactivity of precursors and contributes to the shift in the compressive strength [28]. Moreover, suggestions for the optimal Si/Al ratio vary substantially since some works discuss ratios around 5 [27], while others suggest ratios about 48 [52]. Khalifa et al. [28] also noted the [1] significance of the alkaline activator in the optimization of the mechanical properties. This study reveals better mechanical performance with a lower Si/Al ratio, as depicted in Figure 8. The application of pure hydroxide without the additional soluble silica (contained usually in sodium or potassium water glass) may be a reason for the better mechanical performance [53,54]. Since the sediments or other mineralogically heterogeneous systems contain more than one clay mineral, the selection of the calcination temperature represents an important issue. In other words, dihydroxylation, or recrystallization of kaolinite, illite, and montmorillonite, occurs at various temperature levels, so the applied thermal treatment should also be taken into account within the evaluation of the obtained results. Snellings et al. [36] found that the reactivity of dredging sediments calcinated at 905 °C was unfortunately less reactive compared to metakaolin, which provides a better mechanical performance; however, they may provide better reactivity over siliceous fly ash.

## 4. Conclusions

The potential application of FWS as a precursor for alkali activation presents numerous promising opportunities for construction industries and the future of the building industry. While several materials including coal fly ash or slag cannot be deemed as abundant in the long term in Europe, FWS is a very generous material, often considered a waste product. From this point of view, it offers a sustainable and cost-effective resource for AAMs production. Repurposing FWS to AAMs offers several key benefits: it reduces the demand for natural resources, such as limestone, which are typically required for conventional cement production. This contributes to a circular economy and minimizes the environmental impact of construction activities. Sediment disposal, which is a significant concern in many water bodies, will be addressed, and the burden on landfills and aquatic ecosystems will be reduced. Converting FWS into AAMs requires lower energy consumption compared to traditional cement, leading to potential cost savings. The use of FWS for AAMs can promote regional development by creating opportunities for locally available material production.

Summarizing the obtained results, one can see that the chemical composition of FWS indicates the presence of aluminosilicate in good quantity for alkali activation. The XRD results exhibit the importance of elevated temperature treatment in providing a valuable mineral for AAMs production. The main findings can be summarized in the following points:

- The concentration of the most hazardous elements identified in the studied samples of FWS were very similar compared to alternative cementitious materials such as coal fly ash.
- Since the FWS contains a substantial portion of clay minerals such as kaolinite and illite, the elevated temperature treatment may result in a significant improvement in the material reactivity to meet the requirements for use as an alternative binder system.
- The PSD results present an average range of particle sizes between 27.198 µm and 50.78 µm. This enhanced workability is counterbalanced by a decrease in strength, attributed to the material’s lower reactivity.
- FWS-based AAMs demonstrated sufficient mechanical strength, and a compressive strength of 14.59 MPa to 37.09 MPa was obtained across the samples. This strength enhancement can be attributed to the effective dissolution of aluminosilicate during the alkali activation reaction.
- The development in mechanical performance is related to the content of the amorphous phase and the Si/Al ratio. Specifically, the alkali activation of FWS through the use of potassium water glass and sodium hydroxide provides better performance due to a better-balanced Si/Al ratio and the availability of Na^+^ and K^+^ ions.
- The SEM analysis and MIP results point to the formation of more dense structures for sediments with fine particles, as well as the formation of more rugged surfaces with distinct cracks. More amorphous sediments with lower Si/Al ratios tend to a higher share of pores in the range of 0.01–0.1 µm.

## Figures and Tables

**Table 1:** Composition of mixtures.

Sample	FWS (g)	Potassium Silicate (g)	NaOH (g)	Water (g)	Si/Al Ratio
FWS1	900	280	20	30	4.43
FWS2	900	280	20	35	4.08
FWS3	900	280	20	25	3.97
FWS4	900	280	20	30	4.09
FWS5	900	280	20	45	4.42
FWS6	900	280	20	40	4.58
FWS7	900	280	20	20	3.75
FWS8	900	280	20	55	3.35

**Table 2:** Phase composition of raw sediments.

		A1	A2	A3	A4	A5	A6	A7	A8
Clay minerals	Kaolinite	++	++	+	++	++	++	+	+++
	Chlorite	++	++	+	+	0	+	++	0
	Illite	++	+++	++	++	++	++	+++	++
Quartz		+++	+++	+++	+++	+++	+++	+++	+++
Feldspars	Albite	+	0	0	+	++	+	0	+
	Plagioclase	0	+	+	+	0	0	+	+
	Microcline	+	+	0	++	+	0	0	0
	Anorthite	+	0	0	+	0	0	0	0
Hornblende		+	+	+	0	+	+	0	+
Hillebrandite		+	0	0	+	0	0	+	0

**Table 3:** Oxide composition of studied freshwater sediments after calcination.

Major Oxides (% by Mass)	A1C	A2C	A3C	A4C	A5C	A6C	A7C	A8C
SiO_2_	64.53	66.95	64.02	63.99	63.14	58.59	57.97	60.65
Al_2_O_3_	20.11	18.64	19.96	20.17	20.55	28.57	23.18	22.21
Fe_2_O_3_	4.62	3.84	5.04	5.04	5.4	6.31	8.84	7.45
CaO	1.6	1.7	1.71	1.59	1.66	0.37	1.69	1.28
MgO	2.25	1.85	2.24	2.24	2.31	0.75	1.98	1.89
Na_2_O	1.16	1.48	1.29	1.4	1.25	0.22	0.68	1.07
K_2_O	4.03	3.81	3.54	3.55	3.63	3.37	3.07	2.97
TiO_2_	0.8	0.8	0.9	0.91	0.93	1.04	1.1	1.11
SO_3_	0.26	0.31	0.27	0.35	0.25	0.11	0.39	0.39
P_2_O_5_	0.33	0.36	0.68	0.43	0.54	0.27	0.65	0.57
Cl	0.01	0.01	0	0	0.01	0	0.01	0.01
Sum	99.7	99.75	99.65	99.67	99.67	99.6	99.56	99.6

**Table 4:** Concentration of hazardous elements in studied freshwater sediments after calcination.

Heavy Metals (mg/g)	A1C	A2C	A3C	A4C	A5C	A6C	A7C	A8C
V	0.26	0.25	0.29	0.27	0.28	0.21	0.41	0.36
Cr	0.21	0.16	0.22	0.21	0.22	0.23	0.28	0.26
Mn	1.42	1.34	1.66	1.81	1.41	1.08	2.95	2.51
Co	0.13	0	0.13	0.14	0.12	0	0.16	0.15
Ni	0	0.07	0.06	0.12	0.14	0	0.18	0.13
Cu	0.13	0.12	0.18	0.18	0.22	0	0.2	0.16
Zn	0.76	0.51	0.9	0.75	1	0.22	1.29	1.24
Ga	0.08	0	0	0	0.06	0	0.07	0
Rb	0.39	0.27	0.25	0.28	0.29	0.51	0.27	0.25
Sr	0.33	0.36	0.38	0.4	0.4	0.52	0.27	0.3
Zr	1.02	1.16	1.33	1.14	1.12	2.31	0.67	0.99
Nb	0	0	0.11	0	0.09	0	0	0.09
Ba	1.37	1.45	1.38	1.53	1.72	3.17	1.96	1.77
Pb	0.19	0.17	0.23	0.22	0.2	0.17	0.22	0.2

**Table 5:** Phase composition of calcined sediments.

Material	Amorphous	Quartz	Plagioclase	Anorthoclase	Microcline	Spinel Phase	Maghemite	Ca_3_(PO_4_)_2_	Mica
A1C	8	54	20	3	7	5	0	0	5
A2C	12	49	10	5	8	6	0	5	4
A3C	16	34	11	4	13	7	1	6	10
A4C	18	43	8	9	9	2	0	5	9
A5C	6	49	12	11	8	3	1	3	6
A6C	7	53	9	9	7	4	2	4	6
A7C	10	39	5	18	11	4	1	4	8
A8C	38	28	7	3	11	7	1	3	2

**Table 6:** Basic material properties of alkali-activated FWS.

Sample No.	Bulk Density (kg/m^3^)	Matrix Density (kg/m^3^)	Total Open Porosity (%)
FWS1	1.83	2.48	29%
FWS2	1.86	2.47	24%
FWS3	1.64	2.49	34%
FWS4	1.88	2.49	35%
FWS5	1.89	2.49	24%
FWS6	1.72	2.56	33%
FWS7	1.83	2.53	30%
FWS8	1.86	2.52	26%

## Data Availability

Data are contained within the article.
